# Differences in Immune Response During Competition and Preparation Phase in Elite Rowers

**DOI:** 10.3389/fphys.2021.803863

**Published:** 2021-12-17

**Authors:** Daniel Alexander Bizjak, Gunnar Treff, Martina Zügel, Uwe Schumann, Kay Winkert, Marion Schneider, Dietmar Abendroth, Jürgen Michael Steinacker

**Affiliations:** ^1^Department of Internal Medicine, Division of Sports and Rehabilitation Medicine, University Hospital Ulm, Ulm, Germany; ^2^Department of Anaesthesiology, Division of Experimental Anaesthesiology, University Hospital Ulm, Ulm, Germany; ^3^Department of Surgery, University Hospital Ulm, Ulm, Germany

**Keywords:** rowing, inflammation, stress assessment, immune cells, recovery, load management

## Abstract

**Background:** Metabolic stress is high during training and competition of Olympic rowers, but there is a lack of biomedical markers allowing to quantify training load on the molecular level. We aimed to identify such markers applying a complex approach involving inflammatory and immunologic variables.

**Methods:** Eleven international elite male rowers (age 22.7 ± 2.4 yrs.; VO_2_max 71 ± 5 ml·min^−1^·kg^−1^) of the German National Rowing team were monitored at competition phase (COMP) vs. preparation phase (PREP), representing high vs. low load. Perceived stress and recovery were assessed by a Recovery Stress Questionnaire for Athletes (*RESTQ-76 Sport*). Immune cell activation (dendritic cell (DC)/macrophage/monocytes/T-cells) was evaluated *via* fluorescent activated cell sorting. Cytokines, High-Mobility Group Protein B1 (HMGB1), cell-free DNA (cfDNA), creatine kinase (CK), uric acid (UA), and kynurenine (KYN) were measured in venous blood.

**Results:** Rowers experienced more general stress and less recovery during COMP, but sports-related stress and recovery did not differ from PREP. During COMP, DC/macrophage/monocyte and T-regulatory cells (T_reg_-cell) increased (*p* = 0.001 and 0.010). HMGB1 and cfDNA increased in most athletes during COMP (*p* = 0.001 and 0.048), while CK, UA, and KYN remained unaltered (*p* = 0.053, 0.304, and 0.211). Pro-inflammatory cytokines IL-1β (*p* = 0.002), TNF-α (*p* < 0.001), and the chemokine IL-8 (*p* = 0.001) were elevated during COMP, while anti-inflammatory Il-10 was lower (*p* = 0.002).

**Conclusion:** COMP resulted in an increase in biomarkers reflecting tissue damage, with plausible evidence of immune cell activation that appeared to be compensated by anti-inflammatory mechanisms, such as T_reg_-cell proliferation. We suggest an anti-inflammatory and immunological matrix approach to optimize training load quantification in elite athletes.

## Introduction

In Olympic rowing, a race distance of 2000 m is covered within approximately 5.5–7.5 min, depending on sex, boat class, and environmental conditions and requires a high mechanical power output of 450 to 550 W (Steinacker et al., 1998). To prepare for rowing races, elite athletes spend about 1,128 (1104–1,200) h/year [median (min-max)] for training, corresponding to approximately 23.5 (23.0–25.0) h/week, mainly consisting out of rowing, unspecific endurance and strength training (Fiskerstrand and Seiler, 2004). Rowing training is associated with an outstanding metabolic demand (Winkert et al., 2021) and very different training intensity distributions in international rowing have been reported (Treff et al., 2017, 2021b).

In the light of the training-induced stress, monitoring of internal and external training load is a challenge for scientists, coaches, and athletes (Mäestu et al., 2005; Treff et al., 2021a).

Aside from non-invasive biomarkers like, e.g., resting heart rate, heart rate variability, or mood state, several blood borne variables have been applied to monitor internal training load and/or to diagnose training associated maladaptation (Steinacker et al., 2000; Plews et al., 2014; Hecksteden et al., 2016). These can be categorized into markers of metabolism (e.g., uric acid (UA) and blood lactate), the hormonal system (e.g., cortisol, testosterone, and leptin), and immune response. All of these indicators are known to respond in a highly individual way and due to their specific function in human physiology, and they provide just an incomplete assessment of the individual response to the complex process of acute/long-term training if seen in isolation (Hartmann and Mester, 2000; Podgórski et al., 2020).

Also for this reason, concepts involving inflammation due to cellular damage and metabolic and adrenergic stress inflammatory control gained importance, because they reflect acute and chronic impact of training (Pedersen and Hoffman-Goetz, 2000; Peake et al., 2017). Training, in such a concept, generates inflammatory messages and regeneration promote anti-inflammatory responses (Bay and Pedersen, 2020). Damage-associated molecular patterns (DAMPs) are released into circulation by damaged cells in response to strenuous exercise (Neubauer et al., 2013). DAMPs play a pivotal role for activation and maturation of dendritic cells (DCs), which are important antigen-presenting cells for early immune regulation, recruitment of macrophages, and furthermore T-cell signaling that translates the cellular message to the immunesystem (Brown et al., 2018). It has been shown that the amount of DC increased in rats in response to endurance training without affecting co-stimulatory molecules (CD80/CD86; Liao et al., 2006). Endurance training has also been reported to modulate DC development and to induce a shift toward a more matured state (Chiang et al., 2007). However, there is a lack of studies evaluating the usefulness of DC as an immunologic marker in elite athletes who are exposed to a high metabolic and overall stress. Moreover, the association between circulating and cellular immune variables, DAMPs, and seasonal changes in external training load have, to the best of our knowledge, never been evaluated in elite athletes.

To this end, we aimed to measure the concentration of circulating DAMPs, cytokines, and cell surface expression of cellular immune markers in highly trained male elite rowers at two different phases of a competitive season, specifically during competition phase (COMP; i.e., peak season) and preparation phase (PREP; i.e., low season) while also monitoring mood state. The additional assessment of neurotoxic kynurenine (KYN) should serve as a prospective training load and immune system susceptibility indicator. In doing so, we aimed to establish a basis of immunological and inflammatory variables as well as athletic-specific reference values, potentially serving as training stress markers to monitor training load in elite athletes.

## Materials and Methods

### Patient and Public Involvement

It was not appropriate or possible to involve patients or the public in the design, or conduct, or reporting, or dissemination plans of our research, but the results are intended to support athletes and patients in load/stress management and monitoring.

### Experimental Design and Participants

Eleven highly trained male elite scull rowers, all qualified for the German national team and with top results at international regattas (Table 1) agreed to participate in this study. Two distinct time points were defined, representing high and low competitive stress. The first time point was within the phase of world cup competitions in June, including high intensity training and augmented competitive stress, thereby representing COMP. The second time point was in early November, when rowers had started their preparation period, employing only low-intensity training without any competitions, therefore referred to as PREP. The average weekly training volume was similar between both time points, accumulating to approximately 1,400 min/week and 162 km of rowing. The latest preceding training session consisted of low-intensity endurance exercise and was completed the day before each blood drawing at 12:00 noon at the latest. No high intensity training and/or racing was conducted four days preceding the measurement, in order to minimize the effects of acute exercise in the severe domain. Measurements were made in the morning after getting up. Anthropometric data and performance characteristics of the participants are given in Table 1. Due to the elite status of the participants in terms of physiological characteristics and performance level, it was not possible to create a control group. It was also not possible to divide the existing group, because that would have meant that the control group would not participate in training and competitions. This is not feasible at the level of quasi-professional sport. The study was conducted according to the declaration of Helsinki and approved by the ethical board of the University of Ulm (#267/11). All participants gave informed written consent to participate in the study.

**Table 1:** Anthropometric data and performance characteristics of study participants (*N* = 11, male).

ID	Age (years)	Weight Class M/LM	Standing height (cm)	Body mass (kg)	VO_2max_ (ml/kg/min)	T_2k_ (s)	Results in season 2011
R1	22	M	193	98.7	67	350.7	WCh 2nd; WC 1st
R2	22	M	194	90.4	71	354.9	WCh 2nd
R4	28	M	191	85.5	77	359.6	WC_br_ 1st
R5	22	M	188	86.6	69	356.5	WCh 2nd; WC 2nd
R6	21	M	194	85.2	71	358.8	WC_br_ 3rd
R7	20	M	195	94.6	69	351.3	WCh 2nd; WC 1st
R8	23	M	190	99.2	65	349.7	WCh 2nd; WC 1st
R9	25	M	199	87.7	75	357.6	WCh 2nd; WC 1st
*Mean (M)*	*22.9*		*193*	*91.0*	*71*	*354.9*	
R10	20	LM	*185*	74.3	79	374.8	WCh 4th
R11	25	LM	183	70.5	76	381.1	WCh 8th
R12	22	LM	188	73.0	71	374.2	WCh 4th
*Mean (LM)*	*22.3*		*185*	*72.6*	*75*	*376.7*	
*Mean (total)*	*22.7 ± 2.4*		*191 ± 4.7*	*86.0 ± 9.9*	*72 ± 4.4*	*360.8 ± 10.8*	

*N = 11, male. LM = men, light weight class (≤ 72.5 kg pre-competition weight); M = men, open weight class; t_2k_ = duration of 2000 m rowing ergometer test (Concept 2, Type D); WCh, World Championships; WC, World Cup (Overall Standing); and WC_b_ = World Cup (best ranking at single event)*.

### Recovery Stress Questionnaire for Athletes

To measure stress and recovery perception, the Recovery stress questionnaire for athletes (*RESTQ-76 Sport*) was applied. It covers three days preceding each measurement and has been described in detail elsewhere (Kallus and Kellmann, 2016). Briefly, the *RESTQ-76 Sport* is constructed in a modular way including 48 non-specific and 28 sport-specific items resulting in a comprehensive picture of an athlete’s total stress and recovery. A Likert-type scale is used with values ranging from 0 (never) to 6 (always). The mean of each subtest can range from 0 to 6, with high scores in the stress-associated activity scales reflecting intense subjective strain, whereas high scores in the recovery-oriented items mirror sufficient recovery (Steinacker et al., 2000; Kallus and Kellmann, 2016).

### Blood Sampling

Fasting venous blood was drawn at rest in the morning before breakfast at clinical standard conditions from the *vena brachialis*. Whole blood for flow cytometry was anticoagulated with ethylenediaminetetraacetic acid (EDTA) and stored at 4°C no longer than 30 h prior to fluorescent activated cell sorting (FACS) in order to evaluate immune cell status. Serum, respectively, EDTA-plasma was utilized to assess circulating molecular markers. After transportation at 4–8°C to the laboratory, samples were centrifuged at 2,500 g for 10 min to segregate cells. Supernatants were divided into aliquots to avoid repeated freeze thaw cycles and stored at minus 80°C until the final quantification of analytes.

### Flow Cytometry

Surface markers of whole-blood leukocytes were determined by standard flow cytometric analyses using FACS Calibur and Cellquest software (BD).^1^ Leukocytes were gated into lymphocytes, monocytes, and granulocytes by forward- and side-scatter analysis. Percent-positive cells were quantified *via* direct immunofluorescence staining using fluorescein isothiocyanate (FITC)-conjugated antibodies with phycoerythrin (PE)-conjugated antibodies. After binding of fluorescently labeled antibodies, expression densities of individual antigens were recorded. The expression density of the relevant antigens was calculated as the mean fluorescence intensity (MFI) according to the equation:




MFI=%positives×mean expression density of the relevant antigen−%positives×mean expression density of the respective isotype control

Subsequent monoclonal antibodies were determined separately on monocytes: antibodies directed against HLA-DR (clone L243, BD), CD83 (clone HB15a, Beckman Coulter), and CD123 (clone 9F5, BD). Lymphocytes were evaluated using antibodies directed against CD25 (clone M-A251, BD Biosciences) on its own and together with CD4 (cloneRPA-T4, BD Biosciences), CD2 (clone 39C1.5, Beckman Coulter) either together with CD80 (clone L307.4, Immunotech) or CD86 (clone B-T7; Diaclone). A mouse FITC-IgG1 antibody (clone X40) in conjunction with PE-conjugated IgG2a (clone X39, both from BD Biosciences) served as the isotype controls.

### Plasma Biomarkers

#### ELISA

EDTA-plasma was deployed for the quantification of biomarkers using a highly sensitive and validated ELISA system (Immulite 1,000^®^).^2^ The following Immulite 1,000^®^ kits were used to quantify markers: IL-1β (#6602656), IL-6 (#6604071), IL-8 (#6604136), IL-10 (#6604004), TNF-α (#6602826), and ferritin (#6601889). HMGB1, serving as a marker for necrotic cells, was analyzed using an enzyme immunoassay from IBL-International (ST51011) according to the manufacturer’s manual.

#### Spectrophotometry

##### Uric Acid

A colorimetric endpoint assay (Fluitest UA, Analyticon) was applied for the quantitative determination of UA in serum, where the color intensity is proportional to UA concentration and is analyzed photometrically at 546 nm using a calibrator sample.

##### Creatine Kinase

Circulating total CK in serum was determined using an *in vitro* test from Analyticon (CK NAc, Analyticon^®^ Biotechnologies AG, Lichtenfels, Germany) according to the manufacturer’s manual. Enzyme activity of CK is measured indirectly *via* NADPH amounts. The photometrically measured rate of NADPH formation is proportional to CK and was quantified at 354 nm.

##### Kynurenine

kynurenine was determined by the following protocol: 1000 μm of L-kynurenine sulfate salt (Sigma-Aldrich, Munich, Germany) was diluted 1:20 with 15% Trichloric acid (TCA) to a final concentration of 50 μm. This stock solution was further diluted with TCA to 25 μm, 12.5 μm, 6.25 μm, 3.125 μm, 1.56 μm, and 0.78 μm for standard curve determination. Aqua dest was used as blank solution. 150 μl serum sample was mixed with 100 μl of 30% TCA and centrifuged at 20,000 g for 10 min and 4°C. 150 μl of standard solutions and blank as well as 150 μl of the supernatant were pipetted into a 96-well plate in duplicate and incubated for 15 min at 65°C. 0.15 g of diaminobenzoic acid (DABA) was diluted in 10 ml of 100% acetic acid and 150 μl of this final solution was mixed with the samples for 5 min at room temperature. Fluorescence was measured with a plate reader and the concentration of samples was calculated by the slope of the linear regression line of the optical density from 492 nm minus 620 nm.

##### Circulating Cell-Free DNA Concentration Assay

Cell-free DNA (cfDNA) was determined as described before (Velders et al., 2014). In brief, the cfDNA concentrations in serum samples were directly analyzed with a fluorescent nuclear stain (SYBR Gold) without prior DNA extraction and amplification and a standard curve was generated by serial dilution of commercial salmon sperm DNA. 40 μl of SYBR Gold (1:10,000 dilution in PBS) was added to 10 μl of serum in 96-well plates and fluorescence was recorded using a spectrofluorometer with an excitation wavelength of 485 nm and emission wavelength of 535 nm.

### Statistical Analysis

All data were tested on Gaussian distribution using the Shapiro–Wilk normality test. Pairwise two-tailed t-testing was used for all normally distributed data. Otherwise, a Wilcoxon matched-pairs signed rank test was used to determine statistical significance of differences. For KYN, one-way ANOVA was used to allow for an appropriate multi-group analysis. If significance was observed, Tukey’s multiple comparisons post-hoc test was applied to assess differences between groups (GraphPad Prism, 9.1, San Diego, CA, United States). Statistical significance was established at *p* ≤ 0.05.

## Results

### High Load During Competition Phase Leads to Mental Stress

**Figure 1:**
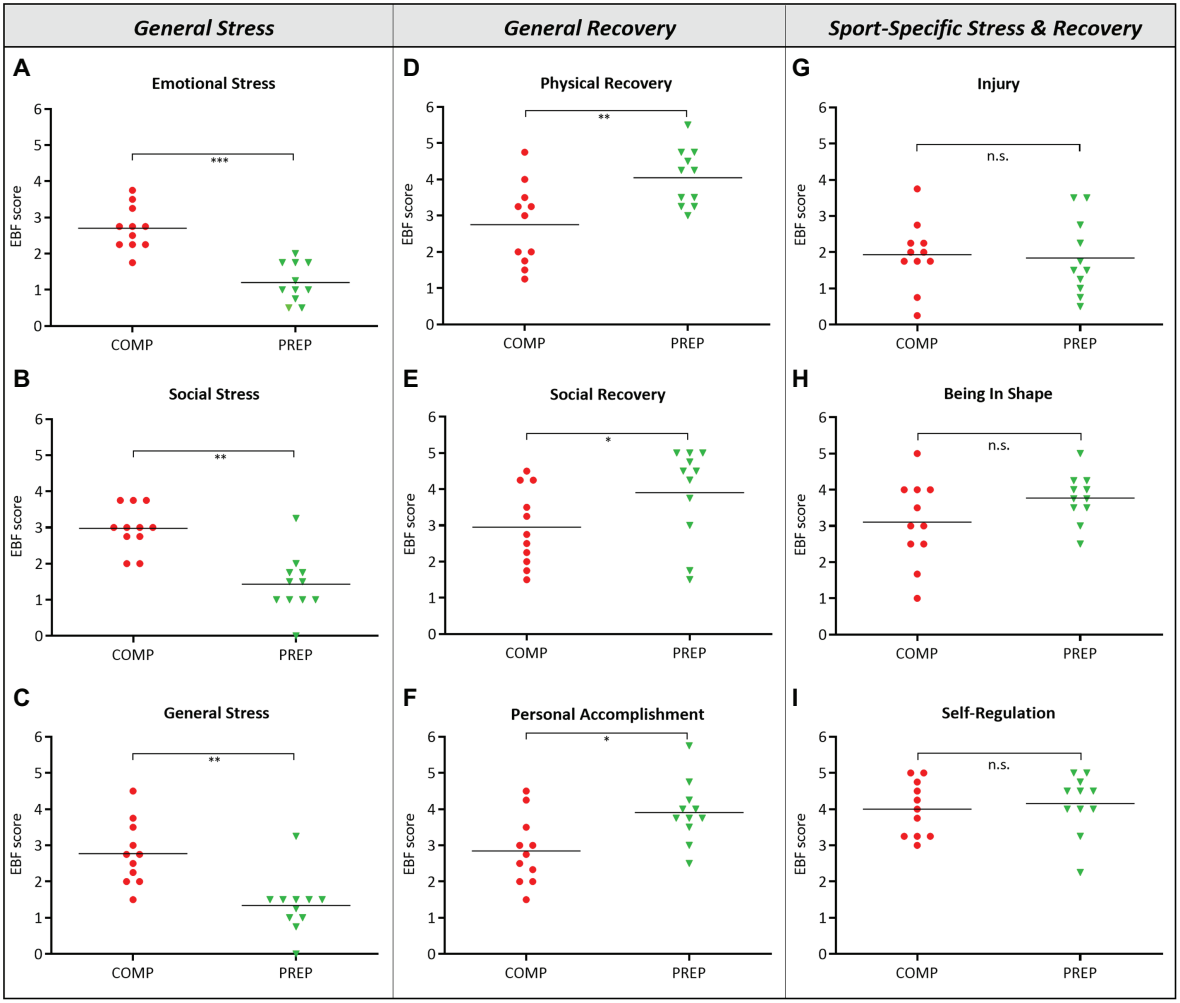
High physical load during competition phase (COMP) in elite rowers induces perceived mental stress. **(A–C)** General stress, **(D–F)** general recovery, and **(G–I)** sport-specific stress and recovery scales and subscales from the RESTQ-Sport questionnaire surveyed during competition and PREP (RESTQ-Sport represents recalls of the last 3 days and answers can range from 0 (never) to 6 (always). ^*^p ≤ 0.05, ^**^p ≤ 0.01, ^***^p ≤ 0.001, and n.s. = non-significant, *N* = 11.

General stress, indicated by the subtests “Emotional Stress” (Figure 1A, *p* < 0.001)‚ “Social Stress” (Figure 1B, *p* = 0.001)‚ and “General Stress” (Figure 1C, *p* = 0.006), were significantly increased during COMP vs. low season. However, even though athletes experienced mental and physical stress, significantly higher scores for the subtests “Physical Recovery” (Figure 1D, *p* = 0.007), “Social Recovery” (Figure 1E, *p* = 0.019), and “Personal Accomplishment” (Figure 1F, *p* = 0.014) during COMP vs. PREP indicated compensatory mechanisms. What is more, despite high general stress loads at COMP, no other significant differences between competition vs. PREP were observed in any subtest representing sport-specific stress and recovery specified as “Injury” (Figure 1G, *p* = 0.834), “Being In Shape” (Figure 1H, *p* = 0.161), or “Self-Regulation” (Figure 1I, *p* = 0.728). This finding is further underlined by a distinct stability of subtests, such as “Conflicts/Pressure,” “Lack of Energy,” “Sleep Quality,” or “Fatigue” (data not shown).

### The DAMPs HMGB1 and cfDNA Are Increased During High and Low Physical Load

**Figure 2:**
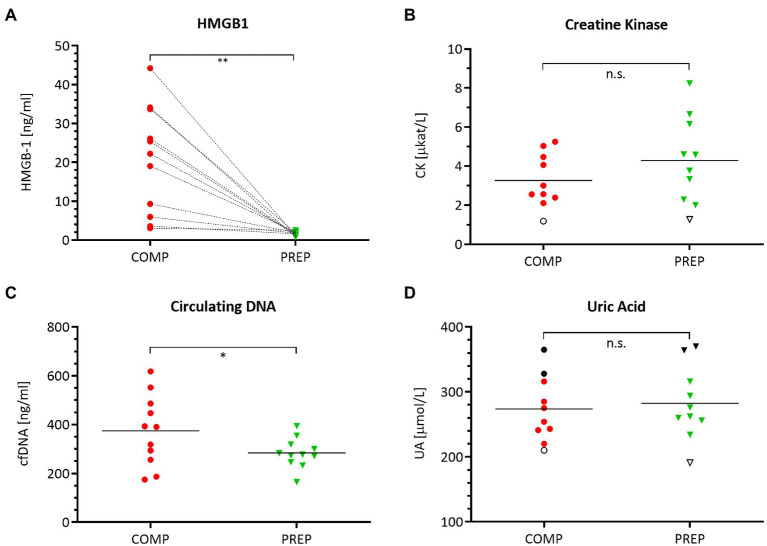
Serum levels of damage-associated molecular pattern (DAMP) molecules **(A)** HMGB1, **(B)** creatine kinase (CK), **(C)** circulating cell-free DNA (cfDNA), and **(D)** uric acid (UA) during competition vs. PREP. ^*^p ≤ 0.05, ^**^p ≤ 0.01, and n.s. = non-significant, *n* = 10 or 11, respectively. HMBG1 is elevated in all athletes compared to low season. Notably, despite a generally large range of most analytes, UA shows a distinct individual pattern as indicated for three athletes mirroring the highest (black circles/triangles) and lowest (empty circle/triangle) UA levels during the course of the season. This also applies in part to CK with an unaltered lowest level of this analyte for a single individual.

HMGB1 (Figure 2A, *p* = 0.0012) and cfDNA (Figure 2C, *p* = 0.048) reflect the elevated physical load of athletes at COMP at resting state and are increased vs. PREP, while the damage marker CK (Figure 2B, *p* = 0.053) and the metabolic stress marker UA Figure 2D, *p* = 0.304) remained largely unaltered.

### Monocyte and Dendritic Cell Activation and Orchestration of Adaptive Immunity

**Figure 3:**
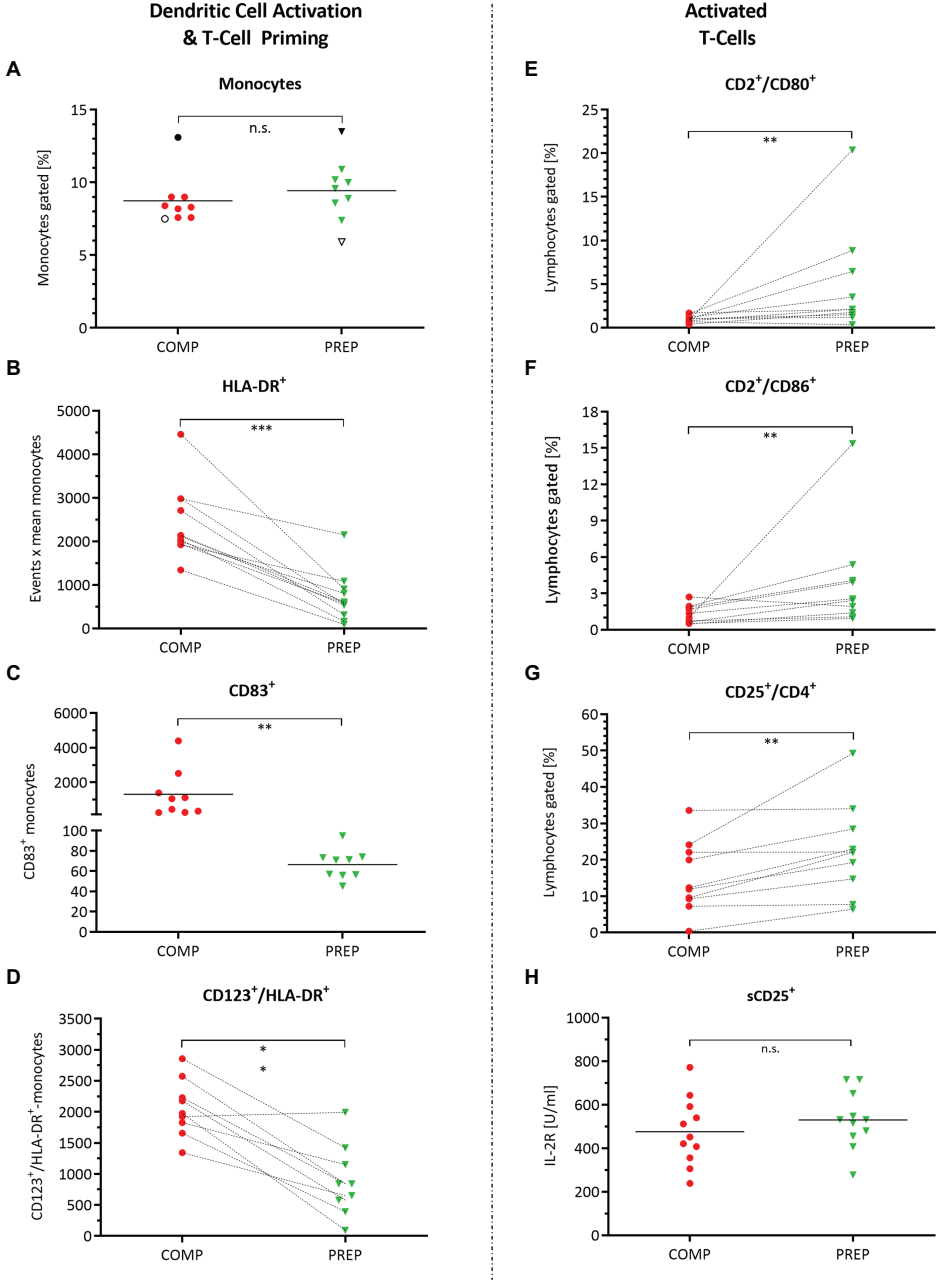
Monocyte and dendritic cell activation and orchestration of adaptive immunity. Cell surface markers detected by fluorescence-activated cell sorting (FACS) quantified in whole-blood cell lysates during competition vs. PREP. ^*^p ≤ 0.05, ^**^p ≤ 0.01, ^***^p ≤ 0.001, and n.s. = non-significant, *n* = 9, 10, or 11, respectively. **(A)** The percentage of monocytes did not differ between competition vs. PREP, whereas increased **(B)** monocyte/macrophage cell surface marker HLA-DR on MΦ detection in COMP indicates immune system stimulation by monocyte/macrophage activation. This is underlined by **(C)** higher measures of CD83^+^ during COMP, a molecule important for antigen presentation, and dendritic cell (DC) maturation and **(D)** CD123, the alpha subunit of the IL-3 receptor, expressed on plasmacytoid DC. Analysis of T-cell activation showed increased values during low season for **(E)** CD2^+^/CD80^+^ and **(F)** CD2^+^/CD86^+^ positive lymphocytes. However, at the same time, **(G)** CD25^+^/CD4^+^ positive immunosuppressive T-regulatory cells increase, suggesting prevention of autoimmune responses. **(H)** Il-2R concentration sorted by sCD25^+^ did not differ between phases.

The percentage of monocytes did not change from competition vs. PREP (Figure 3A, *p* = 0.250); however, the monocyte/macrophage cell surface marker HLA-DR increased (Figure 3B, *p* = 0.001), being a receptor on antigen-presenting cells that are involved in antigen presentation and T-cell priming. In concert with HLA-DR, also CD83 increased (Figure 3C, *p* = 0.004) – which is a molecule important for antigen presentation and DC maturation – as did CD123 (Figure 3D, *p* < 0.001), the alpha subunit of the IL-3 receptor that is expressed on plasmacytoid DC.

Antigen-presenting cells are activated during COMP to orchestrate the adaptive/cellular immune response, involving T-lymphocyte activation to eliminate or prevent pathogen growth. T-lymphocyte activation increased during low season, as indicated by the significant increases in CD2/CD80 (Figure 3E, *p* = 0.006) and CD2/CD86 (Figure 3F, *p* = 0.010) positive lymphocytes. However, at the same time, CD25^+^/CD4^+^ positive immunosuppressive T-regulatory cells increased, suggesting prevention of autoimmune responses (Figure 3G, *p* = 0.010). Il-2R concentration sorted by sCD25^+^ did not differ between competition and PREP (Figure 3H, *p* = 0.822).

### Serum KYN Levels Are Elevated in Patients Suspected to Suffer From Overtraining but Remain Unaltered in Elite Rowers

**Figure 4:**
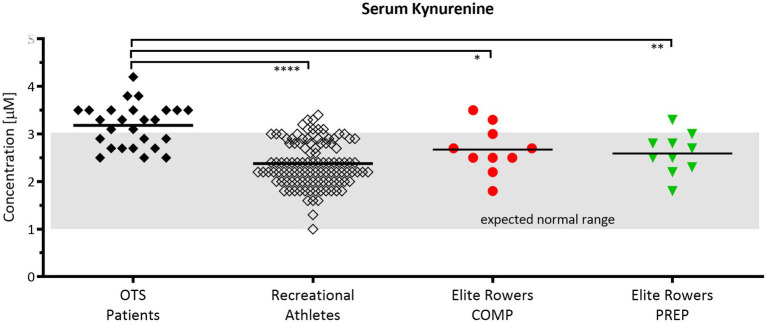
Serum kynurenine (KYN) levels are elevated in patients suspected to suffer from overtraining but are unaltered in response to short periods of overload in elite rowers. Normal range of 1–3 μm according to Kaden et al. (Kaden et al., 2015). Comparison of KYN serum concentrations between patients assumed to suffer from overtraining syndrome (OTS, *n* = 26, black diamond), a group of healthy recreational athletes (*n* = 108, open diamond), and an age cohort of elite rowers during competition and PREP (*n* = 10, red circle vs. green triangle, respectively); ^*^p ≤ 0.05, ^**^p ≤ 0.01, and ^****^p ≤ 0.0001. The grayish rectangle marks the expected normal range of KYN in blood sera of healthy donors (Kaden et al., 2015).

The comparison of KYN serum concentrations (μm) between patients assumed to suffer from overtraining syndrome (OTS), a group of healthy recreational athletes, and an age matched cohort of elite rowers showed that KYN levels between common recreational athletes and rowers at competition vs. PREP are not significantly different among each other, yet considerably different compared to OTS patients (Figure 4). Hence, even competitive world cup stress leads only to slight and non-significant increases of KYN in elite rowers at competition vs. PREP. It is worth to mention that the slight increase in the mean was mainly due to only one individual rower showing an aberrant KYN concentration either at competition or at PREP.

### Circulating Pro-inflammatory Cytokine Concentrations Are Elevated During Competition vs. Preparation Phase

**Figure 5:**
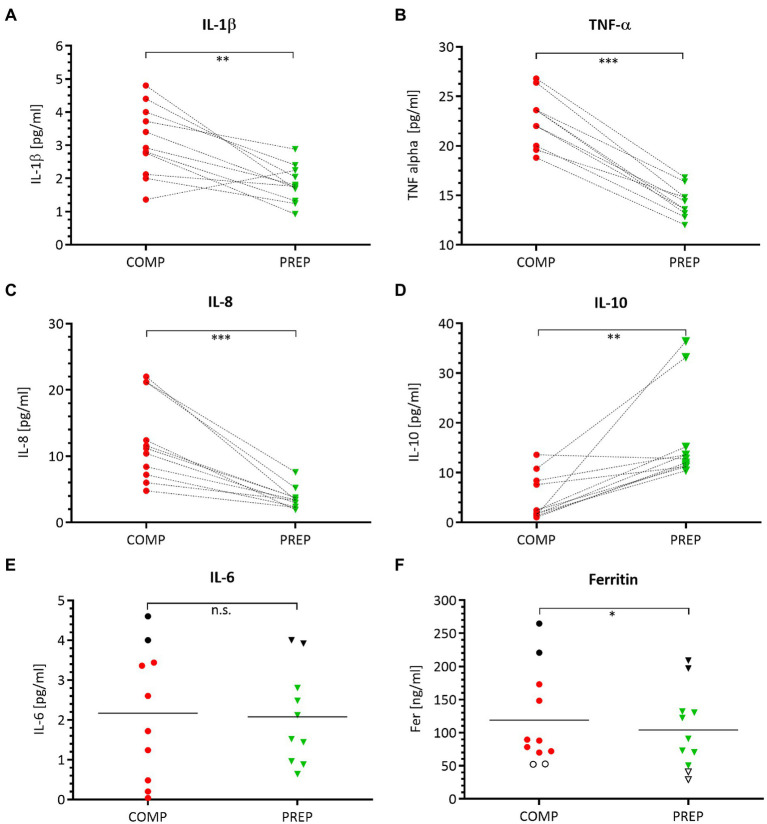
Pro-inflammatory processes are prevailing at COMP, which is illustrated by a distinct cytokine pattern. Serum concentrations of pro-inflammatory cytokines IL-1β **(A)**, TNF-α **(B)**, chemokine IL-8 **(C)**, anti-inflammatory IL-10 **(D)**, acute-phase proteins IL-6 **(E)**, and ferritin **(F)** during competition vs. PREP. ^*^p ≤ 0.05, ^**^p ≤ 0.01, ^***^p ≤ 0.001, and n.s. =non-significant *n* = 10 or 11, respectively.

Serum concentrations of pro-inflammatory macrophage-derived cytokines IL-1β (Figure 5A, *p* = 0.002), TNF-α (Figure 5B, *p* < 0.001), and the chemokine IL-8 (Figure 5C, *p* = 0.001) are elevated at competition vs. PREP. In contrast, the anti-inflammatory cytokine IL-10 (Figure 5D, *p* = 0.002) is significantly downregulated at COMP and shows an increased expression level at PREP. The acute-phase protein Ferritin is lower at PREP (Figure 5F, *p* < 0.050). Noteworthy, the black dots in the upper region of the IL-6 (Figure 5E) and Ferritin (Figure 5F) graphs label two identical rowers at competition, respectively, PREP and indicate that this could be a sign for higher inflammatory stress in these particular athletes, whereas the cytokine/myokine IL-6 shows a nearly unaffected concentration (Figure 5E, *p* = 0.870).

## Discussion

The main results of our study are an increase of psychological and physiological stress in a group of elite rowers at competition vs. PREP, which is accompanied by an increased stimulation of the immunomodulatory response. This response is marked by an increase in damage-associated molecular patterns like cfDNA and HMGB1, and a DC activation indicating an immune response that stimulates the corresponding immune cascade of subsequent T-cell activation and cytokine release.

Athletes experience high psychological and physiological stress during their career, especially in competitive periods (Kellmann and Günther, 2000; Steinacker et al., 2000; Oblinger-Peters and Krenn, 2020). In detail, Steinacker and colleagues reported decreases of performance, gonadal and hypothalamic steroid hormones, and deterioration of psychological recovery during very high load training in elite rowers, thereby highlighting the interaction between mood state and metabolic stress (Steinacker et al., 2000). Our results also indicated an increase in general emotional and physiological stress during COMP in elite rowers, presumably leading to the observed alerted immune response.

Mechanistically, the intense cellular and metabolic stress apparently promote the release of DAMPs from cells into the blood stream, including HMBG1 and cfDNA. HMBG1, normally a DNA-binding protein in the nucleus, serves as a pro-inflammatory cytokine in the cytosol after cellular damage (Scaffidi et al., 2002; Castiglioni et al., 2011; Velders et al., 2014). The HMGB1 increase seen in our athletes with high training load was paralleled by a concomitant increase of cfDNA. This finding confirms similar release kinetics of both cfDNA and HMGB1 observed after short-term treadmill running (Beiter et al., 2011). When such damage and inflammation occur as a consequence of exercise, the released HMBG1 obviously induces the activation and maturation of DCs, a notion that is supported by the significant increases of CD83 and CD123, being indicators of DC activation and maturation (Bates et al., 2015; Feng et al., 2021).

The postulated immune response cascade is in line with other studies reporting highly increased immune response at phases of relatively high training load: Morgado and colleagues reported that after *in vitro* blood immune cell stimulation, DC cell numbers and production of IL-1β, IL-6, IL-12, TNF-α, and MIP-1β decreased in elite swimmers especially early in the season, when training volume increased substantially, potentially compromising the athletes’ immune defense capacities over the whole season (Morgado et al., 2012). Furthermore, secretory patterns of cultured DCs of well-trained skiers and healthy controls revealed that concentrations of several pro-inflammatory cytokines (IFN-α, IL-31, and TNF-β) were higher in the athlete group exposed to training stress (Evstratova et al., 2016). In addition, two animal studies reported an effect of exercise training on rat dendritic cells: Firstly, Liao et al. reported that dendritic cell number increased after training, with no difference in co-stimulatory molecule (CD80 or CD86) expression (Liao et al., 2006), and Chiang et al. found that MHC II expression mixed leukocyte reaction and IL-12 production increased in DCs of exercise trained rats (Chiang et al., 2007). The fact that DC activation is, among other, triggered by cell-free molecules like HMBG1 after cellular damage underlines the potential application of DC activation when it comes to monitoring training-induced stress response.

DC activation in turn may induce further immunomodulatory response with the stimulation and activation of different T-cell and macrophage populations, and the subsequent release of inflammatory cytokines. The increase of pro-inflammatory cytokines IL-1ß, IL-8, and TNF-α observed in our elite rowers suggests an augmented stimulation of the immune system. Since CD25^+^/CD4^+^ expression decreased concomitantly, an increased susceptibility to infections is possible. On the other hand, an increased gating of lymphocytes positive for proteins CD80 and CD86 suggests an upregulated immune response capability at PREP. In general, DCs can activate all types of effector T-cells (T_reg_, T-helper cells, or killer T-cells) and regulate activation and regulation of immune responses (Dieterlen et al., 2016). We did not observe a concomitant increased T-cell activation despite DC activation and T-cell priming COMP, which may be due to insufficient co-stimulatory signals to result in an increased percentage of activated T-cells, potentially indicating a more susceptible immune system.

The reason for this increased susceptibility after intense exercise, reflected in the “open window” theory, is still incompletely understood (Nielsen, 2003; Kakanis et al., 2010). Nielsen et al. examined the impact of a 6-min “all-out” ergometer rowing test on lymphocytes and natural killer (NK) cells. They reported increased NK cell activity and lymphocyte subsets during the test, but while two hours after the rowing test leucocyte and neutrophil numbers remained elevated, the lymphocyte count decreased (Nielsen et al., 1996a,b). Apparently, this acute maximal rowing test did induce lymphocytosis but did not suppress the immune response during recovery, despite the previous involvement of large muscle mass during exercise in the severe intensity domain (Nielsen et al., 1996b), which is also in line with other studies (Nielsen, 2003). However, prolonged and regular intense endurance exercise has been reported to suppress total lymphocyte and NK cell counts as well as neutrophil phagocytic function, suggesting an increased immune system susceptibility during COMP (Kakanis et al., 2010).

Although previous findings associated pro-inflammatory cytokines IL-1ß and TNF-α with depressed mood, sleep disturbances, and stress (Main et al., 2009, 2010), no similar conclusion can be drawn from our data, because sleep and mood state were undisturbed. On the other hand, an overstimulation of the immune system due to sustained high training load may lead to increased susceptibility for pathogen invasion, a mechanism that may be summarized as the open window theory (Nielsen et al., 2016), which is also in line with the lacking T-cell activation mentioned before.

Deregulated or sustained inflammation leads to pathological conditions, such as chronic infection, inflammatory, and autoimmune diseases (Walsh et al., 2011). Also in rowers, acute inflammatory responses to training are associated with decreased aerobic performance (VO_2_max and maximal aerobic power; Jürimäe et al., 2016). In line with that we previously showed that hepcidin and ferritin, both representing acute-phase proteins, are sensitive to initial increases in training load after seven days during a training camp (Zügel et al., 2019). Also, in the present study, we found slightly increased ferritin levels during COMP, which were not associated with significantly increased IL-6 levels. Conversely, this association was reported in a recent review (Larsuphrom and Latunde-Dada, 2021). This contrasting observation may be due to differences in training load assessment or the observed high inter-individual acute-phase reactions in our participants, which reduces the application as possible metabolic stress marker during a whole training season.

Practitioners in the field frequently assess CK or urea to monitor training load, which is also recommended by recent scientific studies and appears to be suitable to measure acute responses (Hecksteden et al., 2016). However, neither CK nor UA differed from competition vs. PREP in our study. In the light of these results, our immunologic approach suggests the promising prospect of better capturing especially cumulated training stress, which is probably at least as relevant for athletic decision making like measures of acute stress.

Another prospective indicator or pathway to monitor training load and immune system susceptibility may be the kynurenine pathway. Kynurenine is a downstream metabolite in the tryptophan metabolism and increased in diseases showing depression disorders (Schlittler et al., 2016). Endurance exercise is a possible treatment for such disorders and was proposed due to the increased metabolization of the neurotoxic kynurenine to its non-toxic form kynurenic acid by kynurenine aminotransferase (Schlittler et al., 2016; Strasser et al., 2016). According to our experience, both immune system and depressive disorders are common in overtrained athletes with concomitantly elevated kynurenine levels (Figure 4). The comparison of our elite rowing athletes’ kynurenine concentrations with either athletes suffering from overtraining or healthy controls showed less abnormal levels in controls and rowers than in overtrained individuals. Finally, these data reveal an necessity for further research, as the normal range was defined by measurements in healthy controls (Kaden et al., 2015) and this range is possibly lower than in elite athletes who often present with abnormal blood and metabolic levels of inflammation and immune responses due to training (Steinacker et al., 1993; Bowen et al., 2011; Banfi et al., 2012).

### Strengths and Limitations

Our study has some limitations worth indicating. The sample size is relatively small, thereby limiting the generalizability of our results. This is, however, an unavoidable problem when studying elite athletes, because an elite group is a small group per definition and furthermore, access and possibility to monitor them is confined. This limitation is scientifically outweighed by the fact that the results of this study are based on training and racing performances that only such an extreme group can realize.

Another limitation is that the data were obtained at only two time points, which is clearly related to the confined access and the relatively high volume of blood drawn per athlete. Notwithstanding, this study underlines the importance of longitudinal monitoring of elite athletes to establish individual normal values/ranges of immunological and inflammatory variables. This is a prerequisite to apply our results to diverse athlete populations and to help optimize athletic performance by improved load management.

### Conclusion and Perspectives

Our data suggest that DAMPs like HMGB1, cfDNA, and particular cytokine matrices are promising markers allowing to monitor especially cumulated training stress in highly trained athletes, and in this respect, they potentially surpass the advantages of conventional indicators. This notion is supported by the concomitant alterations of these markers with training load and the plausibility of the proposed mechanisms. However, individual normal ranges need to be defined due to a substantially high inter-individual variability. In addition, molecular damage markers like mitochondrial DNA (mtDNA; Wenceslau et al., 2014) or heat shock proteins (HSPs; Foell et al., 2007; Wang et al., 2012) may qualify to further improve assessment of internal training load with the ultimate goal to differentiate between functional or non-functional overreaching and the overtraining syndrome, currently lacking validated diagnostic tests and biomarkers (Meeusen et al., 2013; Kreher, 2016). A graphical summary is provided as Supplementary Material.

## Data Availability Statement

The original contributions presented in the study are included in the article/supplementary material, further inquiries can be directed to the corresponding author.

## Ethics Statement

The studies involving human participants were reviewed and approved by the ethical board of the University of Ulm (#267/11). The patients/participants provided their written informed consent to participate in this study.

## Author Contributions

DB, GT, MZ, US, and JS participated in the study design and contributed to data collection and data analysis. MS and DA contributed to sample analysis and experimental protocol evaluation. KW contributed to scientific discussion of the study and the manuscript. All authors contributed to the manuscript writing. All authors have read and approved the final version of the manuscript, and agreed with the order of presentation of the authors.

## Conflict of Interest

The authors declare that the research was conducted in the absence of any commercial or financial relationships that could be construed as a potential conflict of interest.

## Publisher’s Note

All claims expressed in this article are solely those of the authors and do not necessarily represent those of their affiliated organizations, or those of the publisher, the editors and the reviewers. Any product that may be evaluated in this article, or claim that may be made by its manufacturer, is not guaranteed or endorsed by the publisher.

## Footnotes

^1^ biosciences.com/

^2^ https://www.siemens.com/
