# Supplementary material for: Differences in Immune Response During Competition and Preparation Phase in Elite Rowers
Source: Front Physiol. 2021 Dec 17;12:803863. doi: 10.3389/fphys.2021.803863 (PMC8718927; doi:10.3389/fphys.2021.803863)
Supplement: Supplementary file 1 [file Image_1.pdf]

Supplementary Material

Supplementary Figures

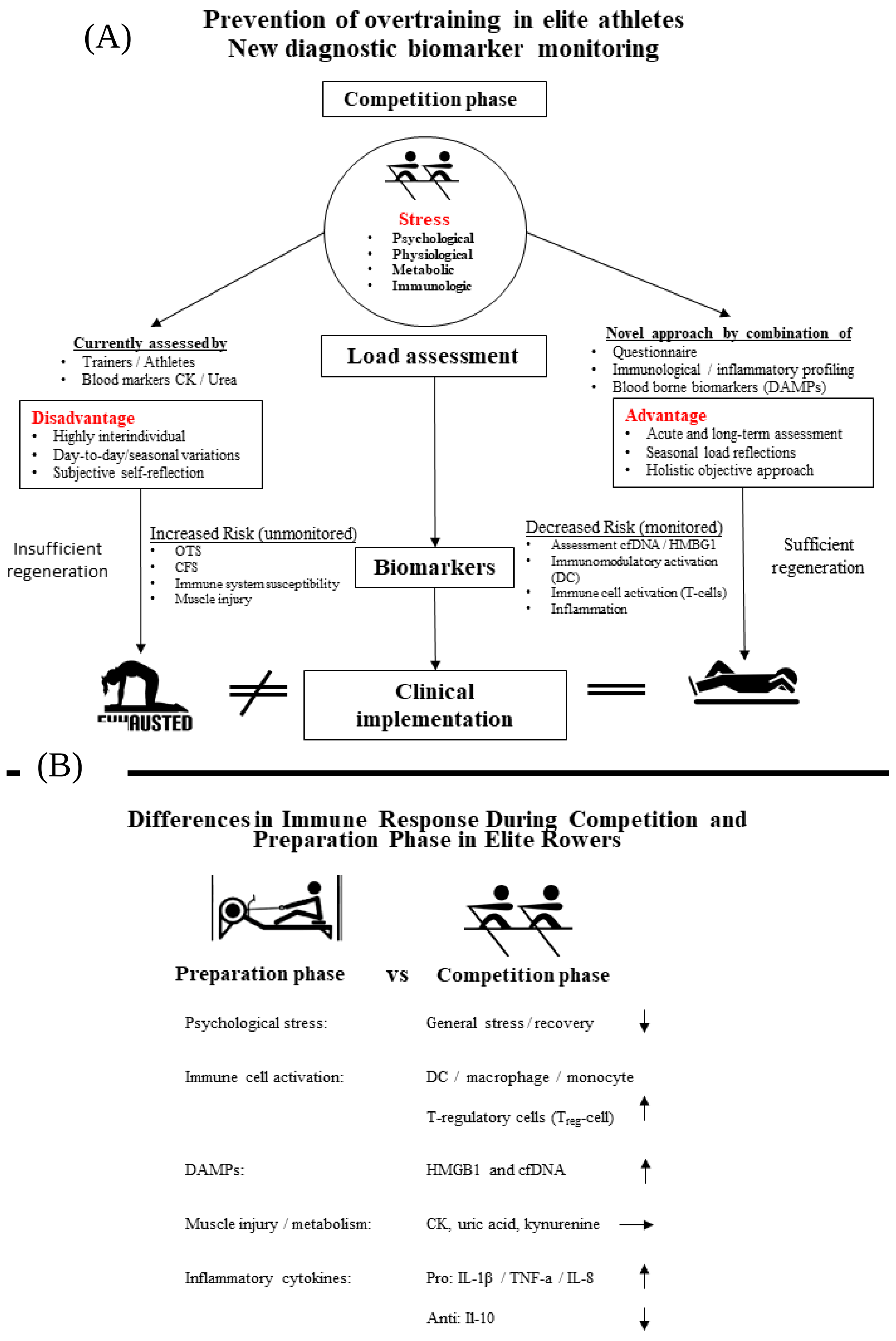

Supplementary Figure 1. Graphical abstract of the study aim (A) and the study results (B). (A) The currently standard and insufficient assessment of training load during a competitive season

potentially leading to overtraining is shown of the left side, while a new approach based on new molecular biomarkers is presented on the right side. (B) Overview of the different psychological and molecular changes in the preparation compared to the competition phase in elite rowers. CK: Creatine Kinase; DC: Dendritic cells; IL: Interleukin; TNF-α: Tumor-necrosis Factor alpha; OTS: Overtraining Syndrome; CFS: Chronic Fatigue Syndrome; DAMP: Damage-Associated Molecular Patterns.
